# Transcriptome-Based Analysis of the Mechanism of Action of Metabolic Disorders Induced by Waterborne Copper Stress in *Coilia nasus*

**DOI:** 10.3390/biology13070476

**Published:** 2024-06-27

**Authors:** Dongyu Huang, Lu Zhang, Haifeng Mi, Tao Teng, Hualiang Liang, Mingchun Ren

**Affiliations:** 1 Key Laboratory of Integrated Rice-Fish Farming Ecology, Ministry of Agriculture and Rural Affairs, Freshwater Fisheries Research Center, Chinese Academy of Fishery Sciences, Wuxi 214081, China; 2 Tongwei Agricultural Development Co., Ltd., Key Laboratory of Nutrition and Healthy Culture of Aquatic, Livestock and Poultry, Ministry of Agriculture and Rural Affairs, Healthy Aquaculture Key Laboratory of Sichuan Province, Chengdu 610093, China

**Keywords:** copper stress, metabolic disorders, immune dysregulation, transcriptome analysis, *Coilia nasus*

## Abstract

**Simple Summary:**

To reveal the effects of waterborne copper stress on gene expression changes, molecular pathways, and physiological functions in *Coilia nasus*, two libraries were constructed from a copper treatment group (Cu) and a control group (C) and sequenced using Illumina sequencing technology. The present study indicates the main view: copper induces the aberrant expression of immune and metabolic aspects of genes, suggesting that copper causes metabolic disorders and insufficient energy supply in the body, and it induces oxidative stress, which results in reduced immune functions.

**Abstract:**

To reveal the effects of waterborne copper stress on gene expression changes, molecular pathways, and physiological functions in *Coilia nasus*, juvenile fish were equally divided into two experimental groups, and the copper levels were 1.61 ± 0.03 mg/L (copper-exposed group) and 0 mg/L (control group), respectively. After 4 h, gill tissue samples were collected for transcript sequencing analysis, and two libraries were constructed from the copper treatment group (Cu) and the control group (C) and sequenced using Illumina sequencing technology. The results showed that approximately 40.2–46.0 M clean reads were obtained from each library, and the percentage of uniquely mapped transcripts ranged from 80.57 to 84.93%. A total of 3915 differentially expressed genes (DEGs) were identified under waterborne copper stress, among which 1300 genes were up-regulated, and 2615 genes were down-regulated. Twelve DEGs were randomly selected for quantitative RT-PCR (qRT-PCR) analysis, and the results confirmed that the transcriptome analysis was reliable. Furthermore, the DEGs were subjected to Gene Ontology (GO) and Kyoto Encyclopedia of Genes and Genomes (KEGG) enrichment analysis, and the results showed that most of the DEGs were involved in metabolic pathways, including steroid biosynthesis, glutathione metabolism, and peroxisome proliferator-activated receptor (PPAR) signaling pathways. Furthermore, due to the waterborne copper levels, *gsk-3β* was significantly up-regulated, while other metabolism-related genes (*tor*, *pi3k*, *lpl*, *aqp7*, *fabp3*) were significantly down-regulated. In addition, the copper-exposed group significantly reduced the expression of some immunity genes (*ifn-γ*, *stat1*, *cxcl10*, and *tgf-β*), and enhanced the expression of *il-1β* and *tnf-α.* In summary, these results indicated that copper causes metabolic disorders and insufficient energy supply in the body, and induces oxidative stress, which results in reduced immune functions.

## 1. Introduction

In recent years, with the rapid development of industry and agriculture, copper (Cu) is widely used as a raw material in the chemical industry, and is discharged in the industrial production process [1]. Copper is an effective component in algaecides and fungicides that are widely used in agricultural production activities. Excessive copper ions in water will cause serious pollution to aquaculture water, and will have strong toxic effects on fish through biological enrichment [2], causing abnormal behavior of fish, physiological function disorders, tissue lesions, and even death [3,4]. On the other hand, after the aquatic products with high copper content enter the food chain, the copper ions in the body will be enriched in the food chain through the enrichment effect, and eventually endanger human health [5]. Therefore, the toxicity of waterborne copper to aquatic animals should be widely concerning.

Gills have many physiological functions such as immune regulation, respiration, osmotic pressure regulation and excretion, and acid–base balance, and are also one of the organs directly in contact with the external environment in aquatic animals [6]. A change in the water environment will directly affect the physiological state of gills, and even aquatic animals. In previous studies, it has been confirmed that gills are directly exposed to heavy metals, so heavy metals accumulate in gill tissue [7]. Common lesions in the gill tissue of the Senegalese sole (*Solea senegalensis*) were observed under copper exposure [8], such as the lamellar fusion and proliferation of epithelial cells, the rupture of capillaries, and the release of red blood cells. Ortiz et al. [9] observed that copper increased gill mucous secretions, hypertrophy of chlorine cells, and blood exosmosis in mummichogs (*Fundulus heteroclitus*). Thus, gills are very sensitive to changes in the physical and chemical properties of water environments; they are not only the main sensing organs of pollutants in water environments, but also provide an important model to evaluate environmental risks in ecotoxicological studies [10].

With the continuous development of omics technology and tools, to deeply understand the molecular regulatory mechanisms of aquatic animals in response to changes in water environments, most researchers adopt omics technology to explore the mechanisms of gill adaptations to environmental stressors [11,12,13] and find effective ways to improve aquatic animals’ responses to adverse environments. So far, more and more studies have been conducted on the transcriptome sequencing analysis of aquatic animal gills under different environmental factors (heavy metal pollutants, hypoxia, salinity, etc.); the expression of *Cu/Zn-sod*, *cat*, *idh1*, *phyh,* and *decr2* in the gill peroxisome pathway is significantly up-regulated by copper stress in red swamp crayfish (*Procambarus cruzii)*, suggesting that the regulatory mechanism is related to the function of oxidative stress [14]. Transcriptome analysis has become a powerful tool for studying the regulatory mechanism of branchial responses to changes in water environments, due to the fact that the results of transcriptome sequencing vary with physiological states and external environments.

*Coilia nasus* is an important migratory fish in the Yangtze River and enjoys the reputation of being one of the “three delicacies of the Yangtze River” due to its nutritional richness. With the breakthrough of artificial breeding and pond breeding technology, *C. nasus* has gradually become a breeding variety with higher economic value [15,16]. Studies have shown that the concentration coefficient of heavy metal copper in Yangtze River sediments is high [17]; although an appropriate amount of copper ions is conducive to the stability of aquatic animals’ internal environments, excessive copper ions will lead to abnormal structures and physiological functions of aquatic animals, and various diseases will occur [18]. So far, no studies have been reported on the immune, metabolic, and oxidative stress responses of *C. nasus* under copper stress. Therefore, this study was aimed at revealing the effects of waterborne copper stress on the gene expression changes, molecular pathways, and physiological functions related to *C. nasus* gills through transcriptional sequencing analysis, so as to provide new thoughts and directions for the healthy culturing of *C. nasus*.

## 2. Materials and Methods

### 2.1. Experimental Materials and Fish

Healthy juvenile *Coilia nasus* (5.0 ± 0.2 g) were collected from the Yangzhong Base of the Freshwater Fisheries Research Center (Zhenjiang, China), and the feeding was stopped 2 days before the formal experiment. 

The copper sulfate (CuSO_4_-5H_2_O) used was analytically pure, and the Cu^2+^ in the water was diluted to the intended concentration (1.68 mg/L) according to the results of previous experiments [19]. The water used in the experiment was water purified after 3 days of aeration, with the following water quality indexes: pH = 7.2, dissolved oxygen >7.0 mg/L, water temperature 25.0 ± 0.5 °C, copper level 1.61 ± 0.03 mg/L (copper-exposed group), 0 mg/L (control group), respectively.

### 2.2. Management Methods

At the beginning of the experiment, the juvenile fish were equally divided into six tanks and divided into two experimental groups (copper-exposed and control), with three tanks of 20 fish in each experimental group. All the juvenile fish were placed in six tanks at the same time, and the behavioral status and poisoning symptoms of the fish were observed immediately during the experiment. After 4 h, when the fish in the copper-treated group showed a near-death state, gill tissue samples were collected from *Coilia nasus* in each experimental group for transcript sequencing analysis. To prevent feeding effects, no bait was fed during the experiment.

### 2.3. RNA Extraction, Library Construction, and Sequencing

This experiment was performed according to the instructions of the Trizol kit (Invitrogen, Carlsbad, CA, USA) for total RNA extraction. RNA quality was determined on an Agilent 2100 Bioanalyzer (Agilent Technologies, Palo Alto, CA, USA). After extraction of total RNA, eukaryotic mRNA was enriched with Oligo(dT) beads. After that, the enriched mRNA was fragmented into short fragments using a fragmentation buffer, and sequencing libraries were prepared after quality control of the RNA samples. The resulting cDNA library was amplified by polymerase chain reaction (PCR) and sequenced by Genetic Variation Biotechnology (Guangzhou, China) using Illumina Novaseq6000 (DynaMax Biotech. Co., Ltd., Shanghai, China). 

### 2.4. Bioinformatics Analysis

First, fastp [20] was used to perform quality control; low-quality data were filtered and clean reads were obtained. After data filtering, the data quality was visualized. Next, HISAT2 (v2.1.0) [21] software was utilized to carry out reference genome-based comparison analysis.

Then, according to the comparison results, Stringtie was used to reconstruct the transcripts [22], and then RSEM was performed to calculate the gene expression [23]. After that, to exclude outlier samples, the expression results of each sample were analyzed using PCA, and Pearson correlation coefficients were calculated between samples as a method to understand the reproducibility of the samples.

### 2.5. Differentially Expressed Genes (DEGs) 

RNAs were analyzed for differential expression between the two groups using DESeq2 (v1.20.0) [24] and edgeR (v3.32.1) [25] software. Differentially expressed genes/transcripts were defined as having a false discovery rate (FDR) parameter below 0.05 and an absolute fold change of ≥2. 

### 2.6. Gene Ontology (GO) and Kyoto Encyclopedia of Genes and Genomes (KEGG) Enrichment Analysis

First, differential genes were mapped to each term in the GO database (http://www.geneontology.org/, accessed on 5 June 2023) and the number of differential genes per term was calculated to count the number of differential genes with some GO function. Pathway significance enrichment analyses were performed in terms of KEGG pathways, and hypergeometric tests were applied to identify GO terms and KEGG pathways that were significantly enriched in differential genes compared to the whole background. 

### 2.7. Real-Time PCR Validation Analysis for Differentially Expressed Genes

Twelve DEGs were selected for RT-qPCR, and the expression levels of the target genes were normalized to those of *β-actin* (forward primer: AACGGATCCGGTATGTGCAAAGC, reverse primer: GGGTCAGGATACCTCTCTTGCTCTG) [26] to validate the results of the RNA-Seq analysis. Total RNA was extracted from the gill tissues of swordfish from control and copper-exposed groups. The extracted RNA was used as a template for quantitative analysis of the genes on a CFX96 fluorescent PCR instrument (BioRad, Shanghai, China) according to the instructions of the HiScript ^®^II One Step qRT–PCR SYBR Green Kit (Vazyme Biotech Co., Ltd., Nanjing, China). The relative expression of each sample was calculated by the 2^−ΔΔCt^ method [27]. Details of the primers are shown in Table 1. 

## 3. Results

### 3.1. Sequence Comparison Reference Statistics 

Table 2 shows the sequencing results of the *C. nasus* gill tissue RNA library; approximately 40.2–46.0 M clean reads of each library were obtained. The percentage of uniquely mapped transcripts ranged from 80.57 to 84.93%, while the percentage of transcripts showing multiple mapping results ranged from 2.63 to 3.79%. 

### 3.2. Sample Relationship Analysis

**Figure 1:**
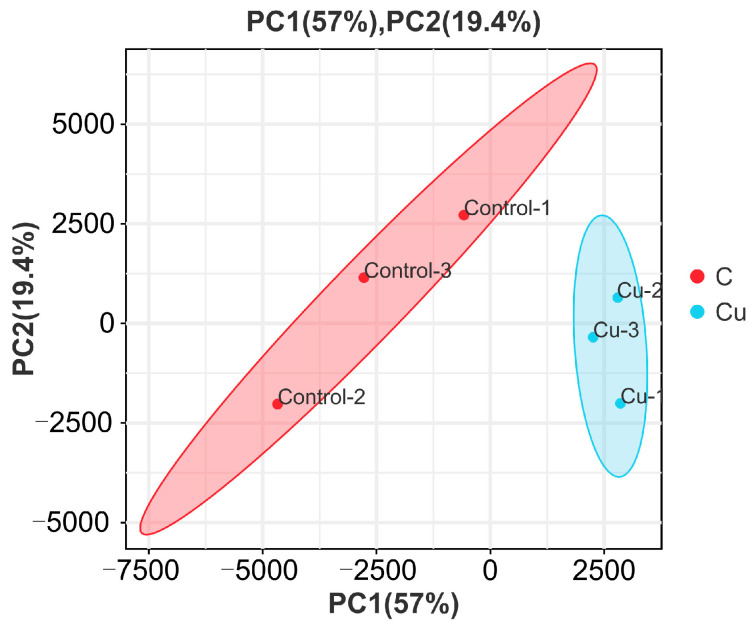
Principal Component Analysis of samples.

**Figure 2:**
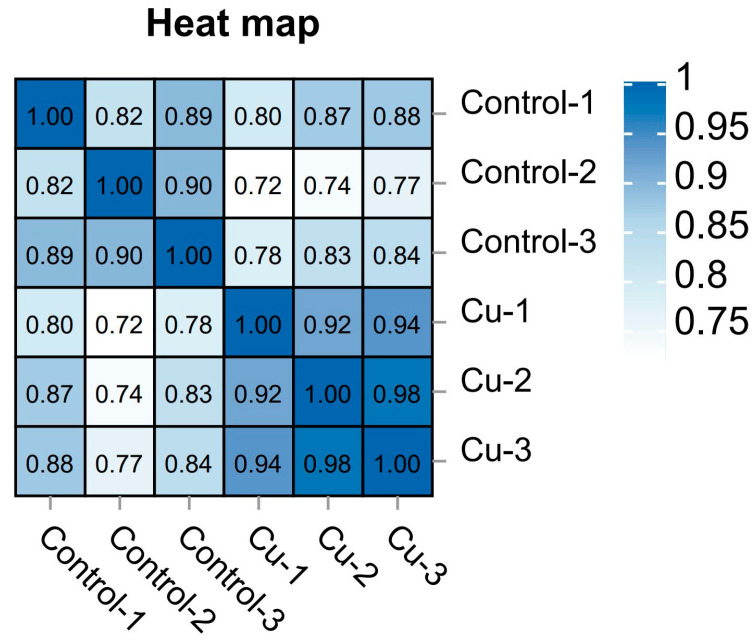
Sample correlation heat map.

Based on the expression volume information, we carried out Principal Component Analysis (PCA). In the analyzed results of the PCA plots, the closer reflected distances represent the more similar sample compositions, and the samples from different effective treatments tend to show their own clustered distribution (Figure 1); it can be seen that the control group is clearly separated from the Cu group in the PCA plot. In addition, the Pearson correlation coefficient visualizes the correlation between any two samples in the form of a heat map, which in our study showed that the repeatability between duplicate samples within groups was good (Figure 2). In the correlation heat map, the correlation coefficients between the three replicates of the control group are all above 0.8, as well as the Cu group. Furthermore, there is a strong positive correlation between samples that belong to different treatment groups, and the lowest correlation coefficient (Cu-1/Control-2) was also greater than 0.72. 

### 3.3. Differentially Expressed Genes (DEGs)

**Figure 3:**
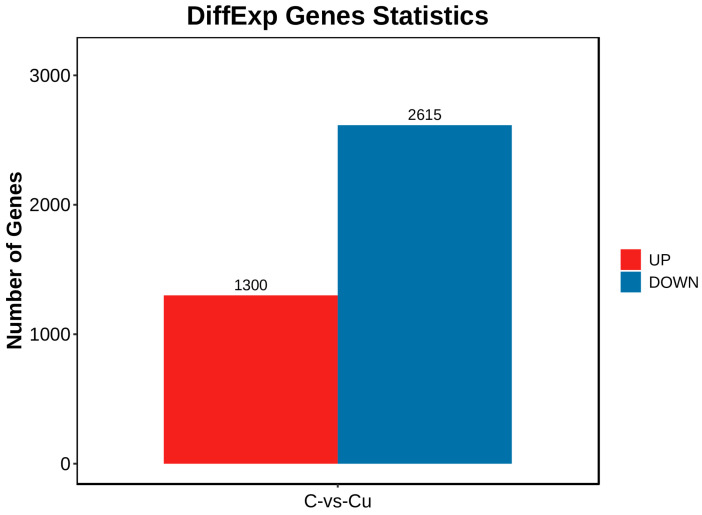
Differential genes statistics chart.

**Figure 4:**
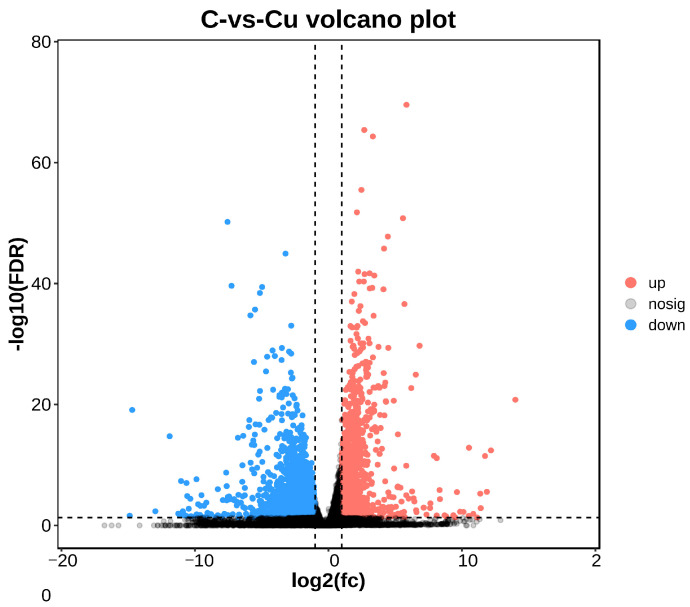
Comparison between C and Cu groups volcano plot.

DEGs between the copper-exposed and control groups (Cu vs. C) were identified; 3915 differential genes (DEGs) were identified in the Cu group, of which 1300 were up-regulated and 2615 were down-regulated (Figure 3). Volcano plots were constructed with DEGs to visualize genes that differed between the two groups, with genes closer to the ends of the plot representing a greater degree of difference (Figure 4).

### 3.4. GO and KEGG Pathway Enrichment Analysis

**Figure 5:**
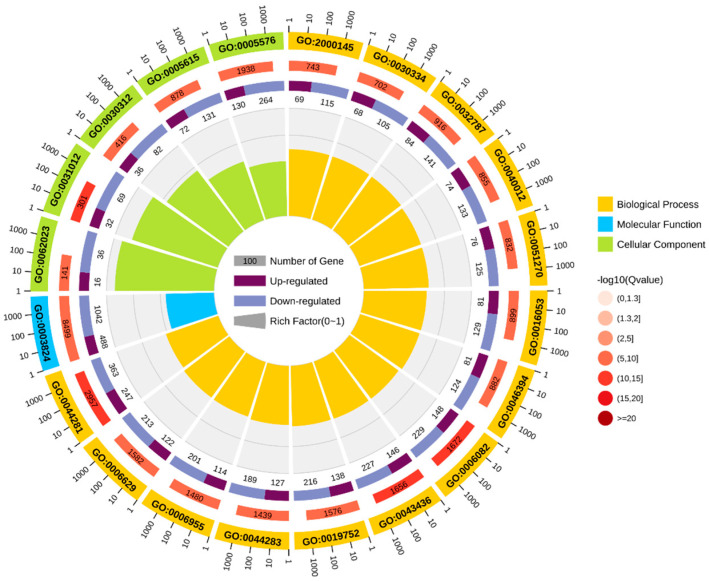
GO enrichment circle diagram.

GO enrichment analysis was performed based on DEGs, and the results were categorized into three categories: cellular components (CCs), molecular functions (MFs), and biological processes (BPs) (Figure 5). Among cellular components, the main categories were the extracellular matrix (GO: 0031012) and external encapsulated structures (GO: 0030312). For molecular functions, the main categories were catalytic activity (GO: 0003824) and small molecule binding (GO: 0036094). For biological processes, the main categories represented are small molecule metabolic processes (GO: 0044281) and organic acid metabolic processes (GO: 0006082). 

**Figure 6:**
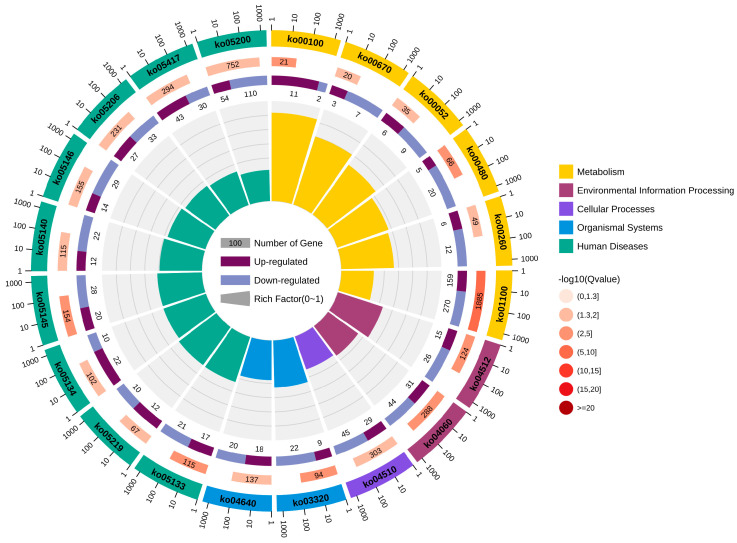
KEGG enrichment circle diagram.

**Figure 7:**
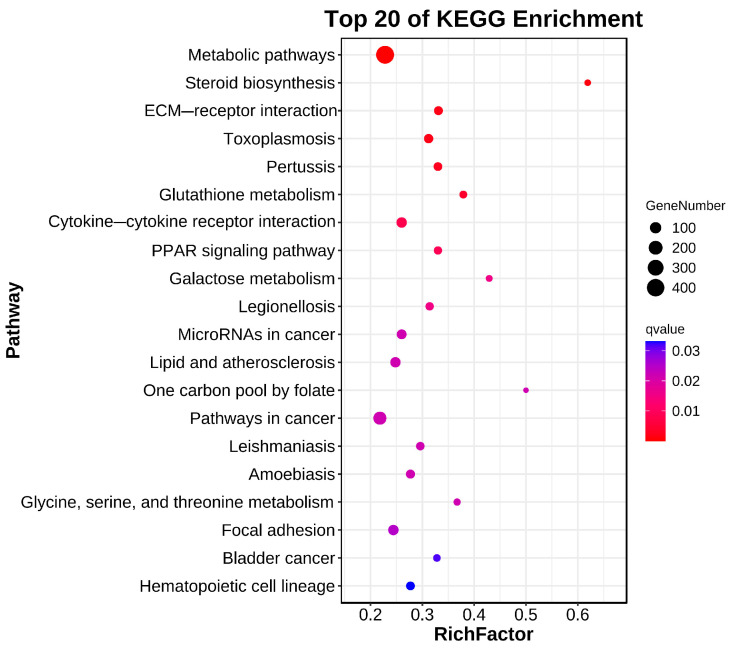
Bubble diagram of KEGG enrichment.

In total, 20 significantly enriched pathways were identified based on KEGG functional annotation (*p-*value < 0.05) (Figure 6). In the Cu and control group comparison, the identified differentially expressed genes were mainly enriched in metabolic pathways (ko01100), steroid biosynthesis (ko00100), ECM receptor interactions (ko04512), glutathione metabolism (ko00480), and the PPAR signaling pathway (ko03320) (Figure 7). 

### 3.5. Validation of RNA-Seq Results with qRT-RCR

**Figure 8:**
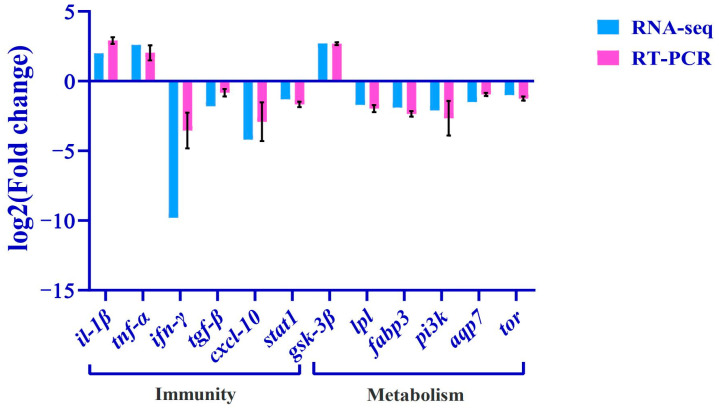
Validation of RNA-Seq results using RT-PCR. The transcript expression levels of the selected genes were normalized to that of the *β-actin*.

Twelve metabolism- or immunity-related differential genes (*il-1β*, *tnf-α*, *gsk-3β*, *ifn-γ*, *pi3k*, *tgf-β*, *cxcl10*, *tor*, *fabp3*, *aqp7*, *lpl*, *stat1*) were selected from the transcriptome database for qRT-PCR validation, and the relative expression levels (expressed as mean ± standard error) of the differentially expressed genes caused by copper stress were quantitatively analyzed using the 2^−ΔΔCt^ method (Figure 8), and the expression trends of the differentially expressed genes were found to be consistent with the quantitative results of the RNA-Seq, which indicated that the results of transcriptome sequencing are reliable.

## 4. Discussion

The irrational discharge of industrial wastewater and the misuse of agricultural pesticides can have a serious impact on the ecosystems of water bodies and threaten the survival of fish [17]. As an indispensable trace element for fish, a high concentration of waterborne copper affects the healthy culture of *C. nasus* [2]. There is an urgent need for transcriptional sequencing technology to reveal the mechanisms of physiological functional changes in *C. nasus* in response to waterborne copper stress. In this experiment, based on PCA and heat map analysis, the Pearson correlation coefficient can reach 0.8 between the three biological replicates within the same group, and the vast majority of gene expression proved that between the three biological replicates in the control and Cu groups, the reproducibility was still relatively good, with no obvious outlier samples, which indicated that the transcriptome results were reliable. In this experiment, the copper-exposed group showed a total of 3915 DEGs compared to the control group, of which 1300 were up-regulated genes and 2615 down-regulated genes. Furthermore, in terms of GO and KEGG enrichment analyses, most differential genes cluster in metabolic pathways including steroid biosynthesis, glutathione metabolism, and PPAR signaling pathway, etc. As reported, after a certain period of copper exposure, it can cause damage to the tissue structure and inhibit the activity of relevant enzymes in the tissues [28,29]. In addition to this, there are also immune pathways (cytokine–cytokine receptor interaction, focal adhesion) involved where some of the inflammatory factors are located. Ma et al. [30] found that copper exposure induces oxidative stress and triggers an inflammatory response in Fugu obscurus (*Takifugu fasciatus*), and Bu et al. [31] reported that copper induces oxidative stress, hepatopancreatic injury, apoptosis, and inflammatory responses in Chinese mitten crabs (*Eriocheir sinensis*). Therefore, it is worth exploring the effects of waterborne copper stress on the molecular pathways and physiological functions of *C. nasus* through transcriptional sequencing analysis, to provide new insights into copper exposure-induced dysfunctions in *C. nasus*.

In recent years, it has been reported that waterborne copper significantly affects lipid metabolism in fish. Studies based on different concentrations of Cu exposure showed that high concentrations of Cu exposure reduced hepatic lipid synthase activity and gene expression in catfish (*Pelteobagrus fulvidraco*), and the lipid contents in the liver and abdominal adipose tissue gradually decreased with the gradual increase in Cu exposure concentration [29]. In this experiment, some DEGs were also enriched in lipid metabolism-related pathways, which in turn resulted in significant changes in the expression of some key genes. Cardiac fatty acid-binding protein 3 (FABP3), whose main function is to bind to intracellular long-chain fatty acids, promotes intracellular long-chain fatty acid transport from the plasma membrane of the cell to the mitochondria, then takes part in β-oxidation in the mitochondria, and ultimately generates ATP, which provides energy for the cell. When *C. nasus* were stressed by copper, the expression of *fabp3* decreased; this implies that avoiding *fabp3* overexpression by decreasing *fabp3* expression will lead to a significant decrease in ATP levels, thus providing a protective effect on the organism [32]. In addition, lipoprotein lipase (LPL) is closely related to adipocyte differentiation and lipid deposition [33], and aquaporin 7 (AQP7) also mediates lipid metabolism in adipocytes [34]. In this experiment, the expression of both *lpl* and *aqp7* was suppressed remarkably; this indicates a reduction in fatty acid uptake and the hydrolysis of triglycerides. Guo et al. [35] reported regarding grass carp (*Ctenopharyngodon idellus*) that the mRNA expression and enzyme activity of lipolytic LPL were significantly decreased after bacterial stress. The increased expression of AQP7 promotes glycerol excretion to the outside of adipocytes and reduces the burden on hypertrophic adipocytes [36]. These results show that lipid has a diminished ability to be oxidized for energy and does not provide more energy to the body.

In addition to lipid metabolism, protein and glucose metabolism were also significantly affected by copper stress. The expression of *pi3k*, which is involved in protein and glucose metabolism, was significantly suppressed. The PI3K/TOR pathway takes part in the regulation of protein synthesis [37]. Phosphatidylinositol 3-kinase (PI3K) can deregulate the inhibitory effect of target of rapamycin (TOR), and activated TOR can phosphorylate S6K, which in turn phosphorylates ribosomal protein S6 kinase, thereby initiating protein synthesis [38]. In this study, copper stress inhibited the *pi3k* and *tor* mRNA expression levels, which in turn inhibited protein synthesis in the organism, resulting in reduced protein deposition. Similar results were also found in swimming crabs (*Portunus trituberculatus*) [39] and the juvenile cobia (*Rachycentron canadum*) [40]. Furthermore, recent studies have revealed that glycogen synthase kinase-3β (GSK-3β) can phosphorylate a variety of endogenous substrates, including multiple proteins and transcription factors involved in metabolism, and, thus, it promotes cell growth and development, and the regulation of glucose homeostasis [41]. Inhibition of GSK-3β activity promotes the dephosphorylation of glycogen synthase, so its activation converts glucose to glycogen, and blood glucose decreases. Conversely, if GSK-3β activity is increased, glycogen synthase activity is inhibited, causing disorders of glucose metabolism [42]. In this experiment, copper stress activated the expression of *gsk-3β* mRNA, and reduced glycogen synthesis, which may lead to insufficient energy reserves in the body. However, further experiments are required to investigate the specific mechanism by which copper stress induces the disruption of glucose metabolism by increasing GSK3β activity.

Interferon γ (IFN-γ) plays important biological functions in immunomodulation, antiviral activities, and the mediation of inflammatory responses [43]. Moreover, signal transducer and activator of the transcription l (STAT1) gene is also involved in the regulation of the type I IFNs signaling pathway and can be induced to be expressed by IFN-γ [44]. In this study, the *ifn-γ* and *stat1* mRNAs were significantly inhibited, which means that cellular immunity and resistance to viral infections began to weaken. As studies have confirmed, similarly to in mammals, IFN-γ in fish has an enhanced ability to clear bacteria [45] and a strong antiviral activity [46]. Furthermore, the downstream signaling molecule *stat1* is also inhibited by copper stress, and dysregulation of this signaling pathway indicates multiple immune system disorders in the body. In addition, interleukin 1β (IL-1β) and tumor necrosis factor α (TNF-α) play important roles in the immune response and are important pro-inflammatory factors [47,48]. In this experiment, *il-1β* and *tnf-α* were clearly activated, which indicated that the body exhibited a pronounced inflammatory response. Further, the up-regulation of transforming growth factor-β (TGF-β) is induced by an increase in inflammatory mediators to suppress the inflammatory response [49], and chemokine 10 (CXCL10) is also associated with inflammatory diseases, autoimmune diseases, an tumors, and has antibacterial immunity when activated [50], but in this experiment, *tgf-β* and *cxcl10* were both decreased significantly by copper stress. This implies that copper stress poses a serious threat to the *C. nasus* organism, with reduced immune functions.

## 5. Conclusions

In summary, transcriptional sequencing indicates that copper stress has profound effects on *C. nasus*. Copper induces the aberrant expression of immune and metabolic aspects of genes, suggesting that copper causes metabolic disorders and insufficient energy supply in the body, and it induces oxidative stress, which results in reduced immune functions. The enrichment of copper in water bodies has a great toxic effect on *C. nasus*, which seriously affects its normal growth and development and physiological functions and may even lead to death; at the same time, the enrichment of copper in water bodies directly reduces the safety of consumption, and indirectly threatens the health of human beings.

## Figures and Tables

**Table 1:** Primers used in the experiment.

DEGs	Forward Primer (5′-3′)	Reverse Primer (5′-3′)	Product Length
*tgf-β*	CTGGAGTCCCAGCACAAGAG	AAGTCGATGTAGAGCGAGCG	107
*tnf-α*	GCTCTTCTGGCCATTGGACT	CTTCAGCCCTCCACCGAAAT	243
*pi3k*	GGCACGACCCACAGAATGTA	GCGAGCAGAGTTATGCAACG	209
*tor*	ACACACTAAGGGTGCTGACG	ATAGATCAAGGCCTGGGGGT	218
*il-1β*	TGAGCCTGAGAGTGCAACTG	AAGTAGCCCTCGAACTTGGC	262
*stat1*	CACACACTGTGAGTTTGCCG	CCGGTAGTGAGGAGGGGTTA	98
*cxcl10*	TCCCACACCATAAAGTGCCC	TGGGCTCCAAGCTAACAGTG	110
*ifn-γ*	GAACCGCTTGGTCATCTGGA	CCGACTCCTGTGCATCTGTT	203
*fabp3*	GGTTGGTGCAGAAACAGCAG	TACAAACGTTCTCACCGCCT	119
*gsk-3β*	ACAACTGGTTTTCGGGGTGT	TCGACCTGACATGCTCCAAC	148
*lpl*	GACTGCGCTTTATGAGCGTG	CCTCCAGCCAGTTGACGAAT	143
*aqp7*	AAGACCCACAGTGGCAGATG	GTAAATAGCAGCACGCAGCC	251

**Table 2:** Sequence comparison reference statistics.

Sample	Total	Unmapped (%)	Unique Mapped (%)	Multiple Mapped (%)	Total Mapped (%)
Control-1	45,755,186	7,188,744 (15.71%)	36,865,026 (80.57%)	1,701,416 (3.72%)	38,566,442 (84.29%)
Control-2	40,201,886	5,650,917 (14.06%)	33,262,081 (82.74%)	1,288,888 (3.21%)	34,550,969 (85.94%)
Control-3	45,713,032	5,819,616 (12.73%)	38,160,705 (83.48%)	1,732,711 (3.79%)	39,893,416 (87.27%)
Cu-1	46,098,414	5,732,074 (12.43%)	39,153,301 (84.93%)	1,213,039 (2.63%)	40,366,340 (87.57%)
Cu-2	44,001,906	7,079,494 (16.09%)	35,726,863 (81.19%)	1,195,549 (2.72%)	36,922,412 (83.91%)
Cu-3	42,779,158	5,988,628 (14.00%)	35,566,904 (83.14%)	1,223,626 (2.86%)	36,790,530 (86.00%)

## Data Availability

Data are contained within the article.
